# Two new species of the bamboo-feeding leafhopper genus *Abrus* Dai & Zhang (Hemiptera, Cicadellidae, Deltocephalinae) from China

**DOI:** 10.3897/zookeys.318.5799

**Published:** 2013-07-26

**Authors:** Lin Yang, Xiang-Sheng Chen

**Affiliations:** 1 Institute of Entomology, Guizhou University, Guiyang, Guizhou, 550025, P.R. China; 2 The Provincial Key Laboratory for Agricultural Pest Management of Mountainous Region, Guizhou University, Guiyang, Guizhou, 550025, P.R. China

**Keywords:** Bamboo leafhopper, Cicadomorpha, distribution, Homoptera, taxonomy

## Abstract

Two new species of the bamboo-feeding genus *Abrus* Dai & Zhang, 2002, *Abrus xishuiensis* **sp. n.** and *Abrus langshanensis* **sp. n.**, are described and illustrated from Guizhou and Hunan, South China. A checklist and a key to 13 known species are given.

## Introduction

The bamboo-feeding leafhopper genus *Abrus* was established by Dai and Zhang (2002) with six species from Hunan, Guangxi, Fujian, Guangdong and Gansu of China (type species: *Abrus hengshanensis* Dai & Zhang, 2002). To date, 11 species are recognized in the genus (Dai and Zhang 2002; Li and Wang 2006; Dai and Zhang 2008; Li et al. 2011) from southern China. Of them, *Abrus brevis* Dai & Zhang, *Abrus coneus* Dai & Zhang and *Abrus leigongshanensis* Li & Wang, were recorded feeding on bamboo (Li et al. 2011).

During on-going studies on species biodiversity of the bamboo-feeding leafhoppers in China, some specimens belonging to undescribed species of the genus *Abrus* were found. The purpose of this paper is to describe two new species and to provide an identification key to the known species of *Abrus*.

## Material and methods

In the present paper, terminology follows Li et al. (2011). Dry specimens were used for the description and illustration. External morphology was observed under a stereoscopic microscope and characters were measured with an ocular micrometer. Measurements are given in millimeters; body length is measured from the apex of the head to the apex of the forewing in repose. The genital segments of the examined specimens were macerated in 10% KOH, washed in water and transferred to glycerine. Illustrations of the specimens were made with a Leica MZ 12.5 stereomicroscope. Photographs of the types were taken with a Leica D-lux 3 digital camera. The digital images were then imported into Adobe Photoshop 8.0 for labeling and plate composition. The type specimens and material examined are deposited in the Institute of Entomology, Guizhou University, Guiyang, China (IEGU).

## Taxonomy

### Abrus

Dai & Zhang, 2002

http://species-id.net/wiki/Abrus

Abrus Dai & Zhang, 2002: 304; Dai and Zhang 2008: 38; Li et al. 2011: 16.

#### Type species.

*Abrus hengshanensis* Dai & Zhang, 2002, by original designation.

#### Diagnosis.

For the diagnosis and relationships of *Abrus* see Dai and Zhang (2008: 38) and Zahniser (2007–present).

#### Distribution.

China (Gansu, Hunan, Fujian, Guizhou, Guangxi and Guangdong).

##### World checklist of species of *Abrus* Dai & Zhang

*Abrus bifurcatus*: Dai and Zhang 2002; China (Guangdong).

*Abrus biprocessus*: Li et al. 2011, Li and Wang 2006; China (Guizhou).

*Abrus breviolus*: Dai and Zhang 2008; China (Zhejiang).

*Abrus brevis*: Dai and Zhang 2002; China (Guangxi).

*Abrus concavelus*: Li and Wang 2006; China (Fujian).

*Abrus coneus*: Dai and Zhang 2002; China (Gansu, Guizhou and Hubei).

*Abrus graciaedeagus*: Li et al. 2011; China (Guangxi).

*Abrus hengshanensis*: Dai and Zhang 2002; China (Hunan).

*Abrus huangi*: Dai and Zhang 2002; China (Guangxi).

*Abrus leigongshanensis*: Li and Wang 2006; China (Guizhou).

*Abrus langshanensis* sp. n.; China (Hunan).

*Abrus wuyiensis*: Dai and Zhang 2002; China (Fujian, Sichuan and Zhejiang).

*Abrus xishuiensis* sp. n.; China (Guizhou).

##### Key to species of the genus *Abrus* (male)

(Modified from Dai and Zhang 2008)

**Table:** 

1	Basal projection of aedeagal shaft shorter than half length of shaft, reduced or absent (Fig. 23)	2
–	Basal projection of aedeagal shaft equal to or longer than half length of shaft (Fig. 11)	3
2	Subgenital plate short, with posterior margin truncate	*Abrus breviolus*
–	Subgenital plate moderately long, with posterior margin rounded (Fig. 19)	*Abrus langshanensis* sp. n.
3	Aedeagal shaft about half length of basal projection, apical appendages extended posterad	*Abrus brevis*
–	Aedeagal shaft as long as or longer than basal projection, apical appendages extended basolaterad (Figs 11, 12)	4
4	Pygofer with one long process at each posterodorsal margin	*Abrus wuyiensis*
–	Pygofer without processes at posterodorsal margin (Fig. 6)	5
5	Pygofer with one pair of processes at each posteroventral margin	*Abrus biprocessus*
–	Pygofer with one process or without processes at each posteroventral margin	6
6	Apical appendages of aedeagus branched at apex (Figs 11, 12)	7
–	Apical appendages of aedeagus not branched at apex	10
7	Apical appendages of aedeagus with small process at base	8
–	Apical appendages of aedeagus without process at base (Figs 11, 12)	*Abrus xishuiensis* sp. n.
8	Pygofer with long process at posteroventral corner; basal projection of aedeagus short, about half length of shaft *Abrus coneus*
–	Pygofer without long process at posteroventral corner, basal projection of aedeagus as long as shaft	9
9	Basal projection of aedeagus with pair of triangular appendages laterally at midlength, aedeagal shaft without ventral flange at apex *Abrus bifurcatus*
–	Basal projection of aedeagus without appendage laterally at midlength, aedeagal shaft with ventral triangular flange at apex	*Abrus concavelus*
10	Pygofer without process at ventral margin	11
–	Pygofer with process at ventral margin	12
11	Apical appendages of aedeagus directed dorsally, basal projection of aedeagus without lateral appendages in ventral view	*Abrus leigongshanensis*
–	Apical appendages of aedeagus directed lateroventrally, basal projection of aedeagus with lateral appendages in ventral view	*Abrus graciaedeagus*
12	Pygofer with process at caudoventral margin; basal projection of aedeagus dentate along ventral margin and with lateral appendages directed ventrally	*Abrus huangi*
–	Pygofer with digitate process in the middle of ventral margin; basal projection of aedeagus with a long process subapically and lateral appendages directed dorsally	*Abrus hengshanensis*

### Abrus xishuiensis sp. n.

urn:lsid:zoobank.org:act:236C1818-2ED6-4197-953E-F64BE108CEF1

http://species-id.net/wiki/Abrus_xishuiensis

Figs 1–12

#### Type material.

Holotype: ♂, **China:** Guizhou, Xishui, Changqiangou (106°12'E, 28°19'N), 700m, on bamboo, 29 Sep. 2000, X.-S. Chen; paratypes: 3 ♂♂, 3 ♀♀, same data as holotype.

#### Etymology.

The species is named after the type locality, Xishui, Guizhou Province in China.

#### Description.

**Measurement.** Body length including forewing male 9.02–9.25 mm (N = 4), female 9.35–9.90 mm (N = 3); forewing length male 7.62–8.10 mm (N = 4), female 8.00–8.40 mm (N = 6).

**Figures 1–12.**
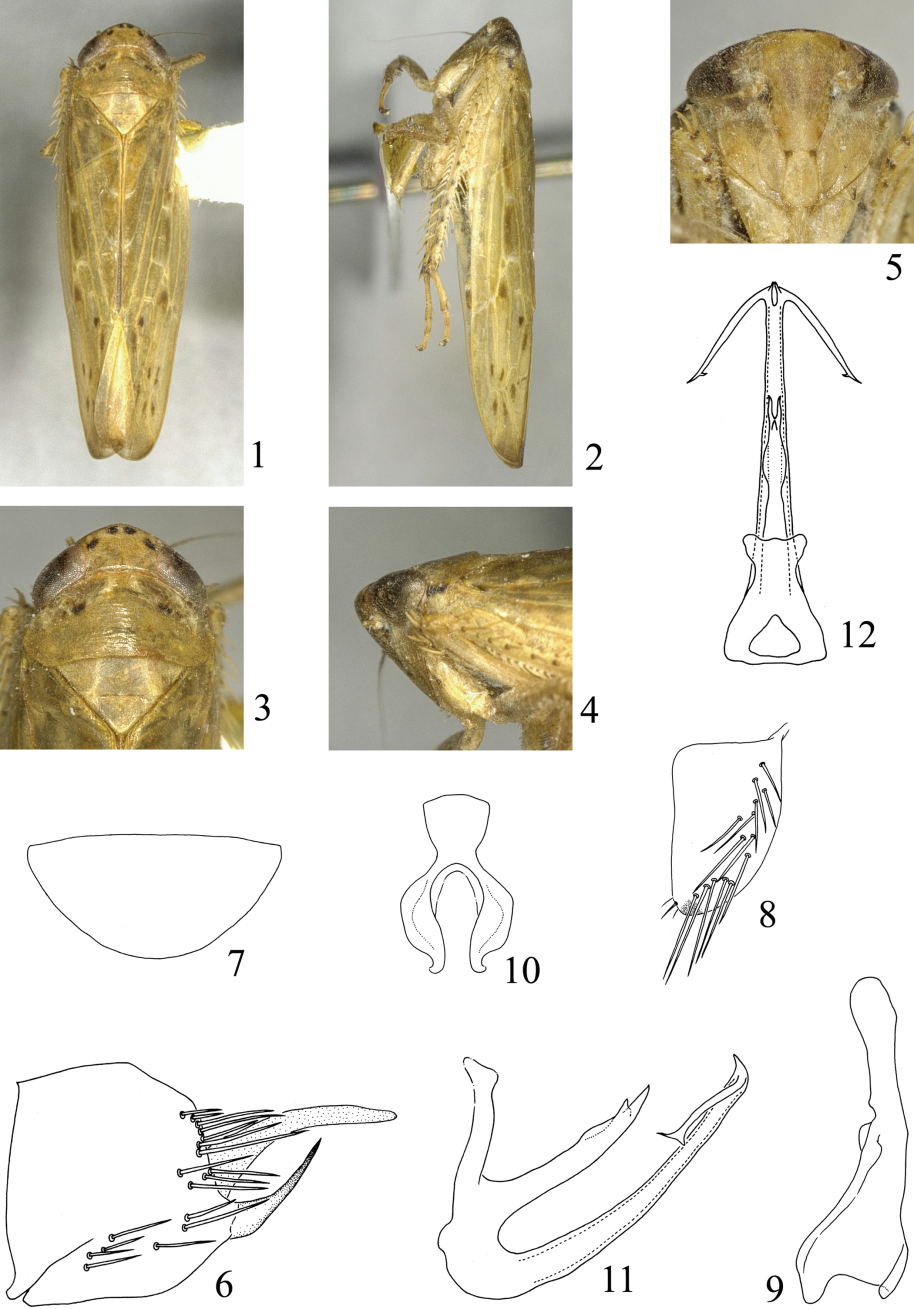
*Abrus xishuiensis* sp. n. **1** Male habitus, dorsal view **2** Same, lateral view **3** Head and thorax, dorsal view **4** Same, lateral view **5** Face **6** Pygofer, lateral view **7** Valve **8** Subgenital plate **9** Style **10** Connective **11** Aedeagus,lateral view **12** Same, posteroventral view.

**Coloration.** Orange to yellowish brown (Figs 1–5). Crown with two pairs of similar blackish brown spots on anterior margin, along suture pale reddish orange. Eyes blackish brown, anterior angle pale reddish brown. Pronotum with pair blackish brown spots on anterior part, with short pale reddish orange stripe centrally. Scutellum with reddish orange marking centrally, transverse suture pale reddish orange. Inner and central anteapical cells at apex, third and fourth apical cells at base each with a dark brown spot.

**Head and thorax.** Crown length 0.7× medial width between eyes. Pronotum length 1.93× medial length of crown. Scutellum length 0.93× medial length of pronotum. Forewing length 3.87× medial width at widest part.

**Male genitalia.** Pygofer (Fig. 6) trapeziform in shape, with macrosetae along posterior margin and midventrally; posterior margin truncate; posteroventral process broad at base, acute apically, slightly curved dorsad, directed posterodorsad; membranous process at inner apex, slightly curved ventrally, broad at base, acute apically, apex acute. Genital valve (Fig. 7) broad triangular, posterior margin rounded, basal width 2.02× median length. Subgenital plate (Fig. 8) broad and short; outer margin rounded; with many macrosetae on lateral region. Style (Fig. 9) long; broad at base; narrow at middle; apex slightly widening; apical margin rounded. Connective (Fig. 10) Y-shaped, shaft robust, arms well developed, shaft length 0.65× length of arm. Aedeagus (Figs 11, 12) with developed basal projection dorsally, about 2/3 length of aedeagal shaft; apex branched in dorsal view; dorsal margin with a stout tooth subapically, grooved at apical third; aedeagal shaft in profile (Fig. 11) slightly curved dorsad, slender, long, tapering apically; dorsal margin of apex with pair small processes, beak-like, directed dorsally; shaft with pair of lateral appendages subapically, each with apex branched. Phallotreme apical on ventral surface.

#### Host plant.

Bamboo (*Chimonobambusa angustifolia* C. D. Chu & C. S. Chao).

#### Distribution.

Southwest China (Guizhou).

#### Remarks.

This species resembles *Abrus biprocessus* Li, 2011 in appearance, but can be distinguished by body size ♂ 9.02–9.25 mm, ♀ 9.35–9.90 mm (♂ 8.1 mm, ♀ 8.2 mm in *biprocessus*); pygofer with one process at posteroventral corner (with two processes in *biprocessus*); basal projection of aedeagus with apical appendages stout and short (slender and long in *biprocessus*); subapical appendages of aedeagal shaft branched at apex, without a small branch basally (not branched at apex, with a small branch at basal third in *biprocessus*).

### Abrus langshanensis sp. n.

urn:lsid:zoobank.org:act:EDFF6A6F-7337-4AAE-915F-FB2631FDE451

http://species-id.net/wiki/Abrus_langshanensis

Figs 13 –27

#### Type material.

Holotype: ♂, **China:** Hunan, Xinning, Langshan (110°49'E, 26°22'N), on bamboo, *Indocalamus* sp., 6 Oct. 2010, X.-S. Chen and L. Yang; paratypes 1 ♂, 1 ♀, data same as holotype; paratype 1 ♂, Hunan, Xinning, Langshan, on bamboo, *Indocalamus* sp., 2 Oct. 2011, X.-S. Chen and L. Yang.

#### Etymology.

This species is named after the type locality, Langshan, Xinning, Hunan Province in China.

#### Description.

**Measurement.** Body length including forewing male 9.10–9.60 mm (N = 3), female 9.55 mm (N = 1); forewing length male 7.70–8.10 mm (N = 3), female 7.85 mm (N = 1).

#### Coloration.

**Figures 13–23.**
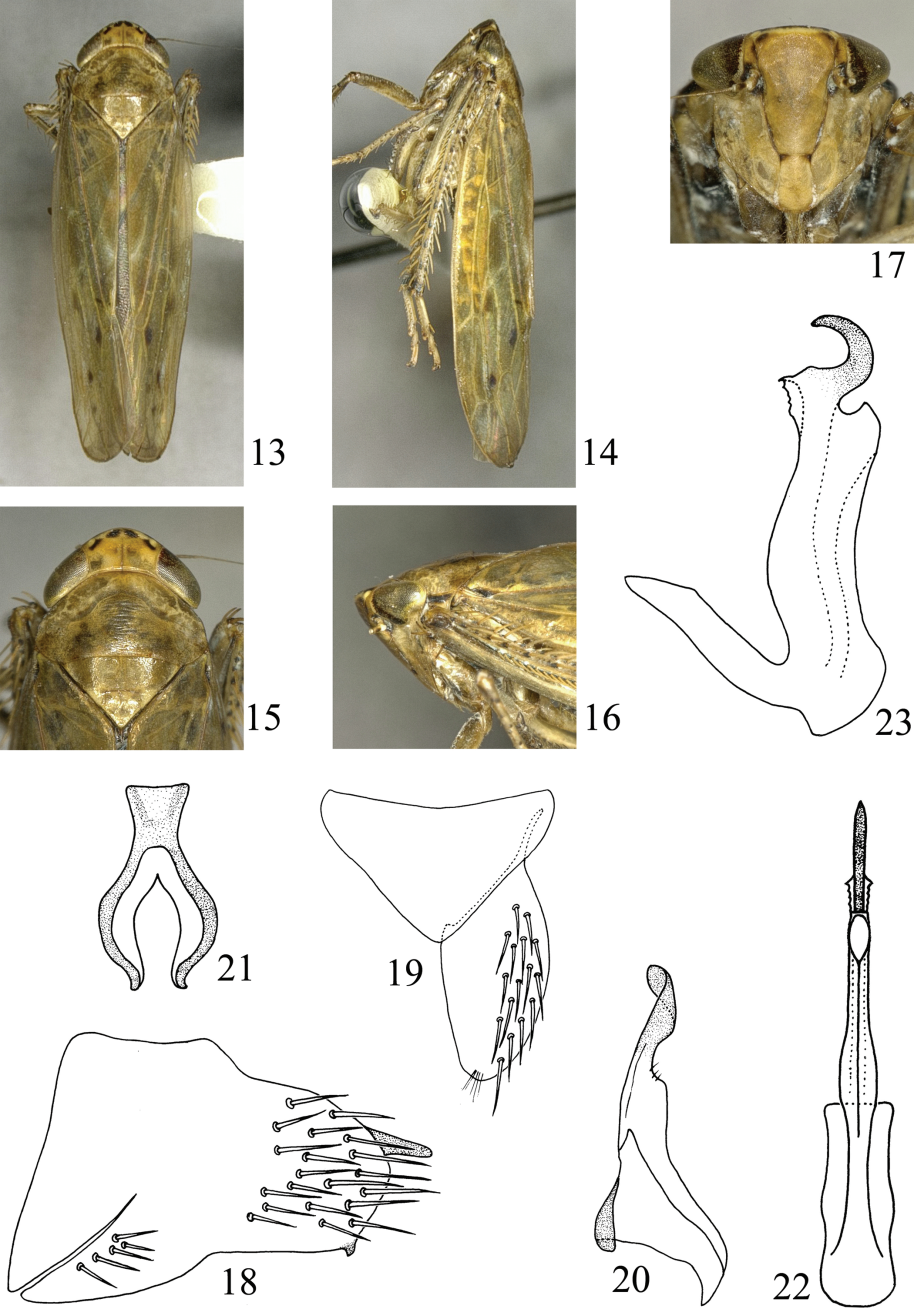
*Abrus langshanensis* sp. n. **13** Male habitus, dorsal view **14** Same, lateral view **15** Head and thorax, dorsal view **16** Same, lateral view **17** Face **18** Pygofer, lateral view **19** Valve and subgenital plate **20** Style **21** Connective **22** Aedeagus, posteroventral view **23** Same, lateral view.

**Measurement.** General color pale yellowish orange (Figs 13–17). Crown with two pairs of similar blackish brown spots on anterior margin. Eyes blackish brown, ocelli pale yellowish white. Face pale yellowish white. Inner and central anteapical cells at apex, third and fourth apical cells at base each with a dark brown spot.

**Head and thorax.** Crown medial length 0.58× width between eyes. Pronotum length 2.03× medial length of crown. Scutellum length 0.87× medial length of pronotum. Forewing length 4.00× medial width at widest part.

**Male genitalia.** Pygofer in lateral view (Fig. 18) narrower posteriorly; covered with macrosetae posteriorly, with several basoventrally dorsad of ventral margin; dorsal margin sinuate; ventral margin concave medially; with a small papillae posteriorly. Genital valve (Fig. 19) broad triangular; posterior margin slightly acute and rounded; basal width 1.95× medial length. Subgenital plate (Fig. 19) broad and short; outer margin roundedly curved; with many macrosetae on lateral region. Style (Fig. 20) long; broad at base; narrowing apically; apex slightly recurved; apical margin acute and rounded. Connective (Fig. 21) Y-shaped; stem robust; arms well developed; length of stem 0.44× that of arm. Aedeagus (Figs 22, 23) without basal projection dorsally; aedeagal shaft in lateral view (Fig. 23) almost S-shaped; apex produced into a robust hook-like process. Phallotreme apical on ventral surface.

#### Host plant.

Bamboo (*Indocalamus* sp.).

**Figures 24–29.**
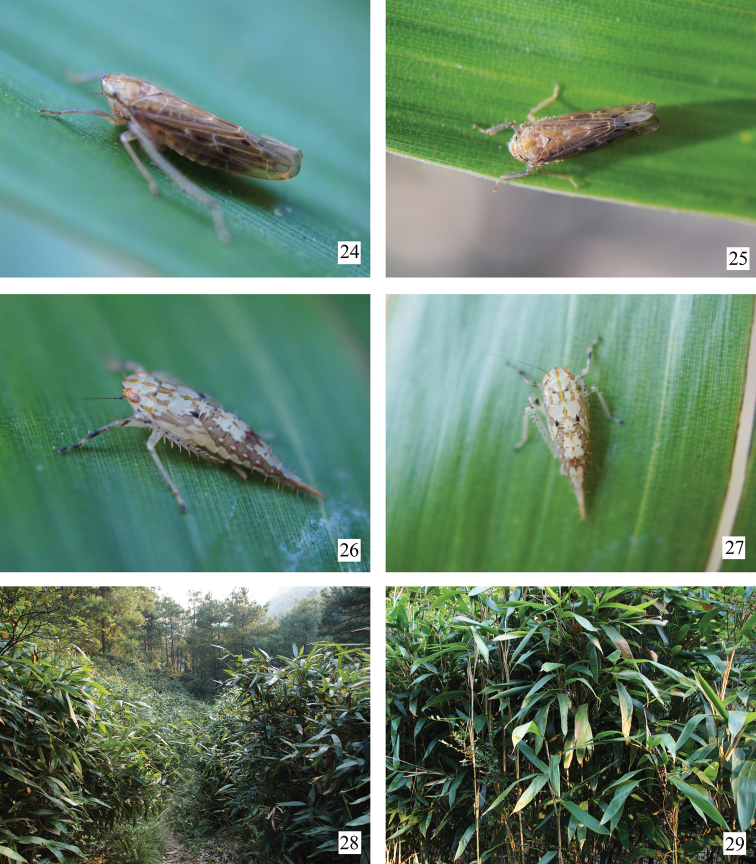
*Abrus langshanensis* sp. n. and its host plant. **24, 25** Adult resting on leaf of bamboo **26, 27** Nymph resting on leaf of bamboo **28** View of the area where *Abrus langshanensis* were captured, in Langshan, Xinning, Hunan, with *Indocalamus* sp. **29** View of the plant. Photograph by X.-S. Chen.

#### Distribution.

South China (Hunan).

#### Remarks.

This new species is similar to *Abrus breviolus* Dai & Zhang, 2008 in aedeagus having reduced or small basal projection dorsally, but can be distinguished by posterior margin of male pygofer without process (with stout process dorsally in *breviolus*); apical margin of subgenital plate rounded (truncate in *breviolus*); aedeagal shaft with apex hook-like, without pair of subapical appendages (with pair of subapical appendages laterally in *breviolus*).

## Supplementary Material

XML Treatment for Abrus

XML Treatment for Abrus xishuiensis

XML Treatment for Abrus langshanensis
